# Pairing transcutaneous vagus nerve stimulation with an intensive bimanual training in children and adolescents with cerebral palsy: study protocol of a randomized sham-controlled trial

**DOI:** 10.3389/fneur.2024.1441128

**Published:** 2024-08-16

**Authors:** Viola Oldrati, Verusca Gasparroni, Arianna Michelutti, Andrea Ciricugno, Renato Borgatti, Simona Orcesi, Elisa Fazzi, Alessandra Morandi, Jessica Galli, Luigi Piccinini, Cristina Maghini, Maria Arioli, Zaira Cattaneo, Cosimo Urgesi, Alessandra Finisguerra

**Affiliations:** ^1^Scientific Institute, IRCCS E. Medea, Bosisio Parini (LC), Italy; ^2^Department of Brain and Behavioral Sciences, University of Pavia, Pavia, Italy; ^3^IRCCS Mondino Foundation, Pavia, Italy; ^4^Department of Clinical and Experimental Sciences, University of Brescia, Brescia, Italy; ^5^Unit of Child Neurology and Psychiatry, ASST Spedali Civili of Brescia, Brescia, Italy; ^6^Department of Human and Social Sciences, University of Bergamo, Bergamo, Italy; ^7^Laboratory of Cognitive Neuroscience, Department of Languages and Literatures, Communication, Education and Society, University of Udine, Udine, Italy

**Keywords:** cerebral palsy, transcutaneous vagus nerve stimulation, neuromodulation, HABIT-ILE, upper limbs motor deficits

## Abstract

**Background:**

Gross motor function impairments and manual dexterity deficits are frequently observed in children and adolescents with Cerebral Palsy (CP), having a major impact on their activity level and autonomy. Improving manual dexterity and activity level of patients with CP is often the focus of rehabilitation. Novel and adjuvant treatment methods that could support the standard training also in chronic conditions are a research priority. The transcutaneous Vagus Nerve Stimulation (tVNS) is a non-invasive brain stimulation technique, which provides a bottom-up stimulation of subcortical and cortical brain structures, enhancing brain GABA and Noradrenaline levels. This technique may play a pivotal role in brain plasticity, which has not been tested in CP patients before.

**Methods:**

44 children and adolescents with CP will be involved, treated in pairs in a randomized, double-blind, pre-post test study. The two groups will undergo the Hand-Arm Bimanual Intensive Therapy Including Lower Extremities (HABIT-ILE) for 2 consecutive weeks, with 3 h daily sessions for 5 days per week, for an overall time interval of 30 h; the training will be combined with the application for 75 min/day of active or sham tVNS, in separate, randomly allocated groups. The primary outcome measure will include the scores at the Assisting Hand Assessment and Box and Block Test, and at an *ad-hoc* visuomotor task evaluating manual visuomotor control. Secondary outcomes will include the scores at the Children’s Hand Experience Questionnaire, Canadian Occupational Performance Measure, Melbourne Assessment of Unilateral Upper Limb Function, Gross Motor Function Measure, Vineland, Pediatric quality of life inventory. The evaluation points will include pre (T0), post (T1) and 3-month follow up (T2) assessments. Safety and tolerability will also be assessed.

**Results:**

The results of this trial will assess whether tVNS can effectively boost the effects of an intensive two-week bimanual training, in improving manual dexterity in children and adolescents with cerebral palsy, ensuring safety and tolerability throughout the intervention period.

**Clinical trial registration**:  ClinicalTrials.gov, NCT06372028.

## Highlights

- Enhancement of the effectiveness of intensive bimanual training.
- Set of best practice parameters of tVNS in pediatric patients.
- Proof of safety and tolerability of tVNS in the CP population.

## Introduction

Intrauterine or neonatal encephalopathy, whether vascular, infectious, or neuroinflammatory in origin, is a cause of brain destruction and reorganization and can lead to serious, debilitating conditions, including neurodevelopmental impairments such as Cerebral Palsy (CP) (1). The term of CP describes a group of permanent disorders compromising movement and posture, causing activity limitation, often accompanied by disturbances of sensation, perception, cognition, communication, and behavior. These difficulties directly impact social participation and quality of life of children and their families until adulthood (2, 3), with an overall prevalence of 2.11 per 1,000 live births (4, 5).

Depending on brain lesion location, either bilateral or unilateral CP may be observed. Upper limb functionality has a huge impact on a child’s ability to carry out self-care tasks and participate in leisure and learning activities. Indeed, patients often experience frustration when attempting to perform everyday two-handed tasks, and they display greater negative reactions to failure than their typically developing peers (6).

While there is no cure, there is a wide consensus in the literature on the importance of an early and intensive intervention to alleviate negative impact. Many different approaches based either on the training of a motor ability or the training of a task to achieve a goal or to acquire motor skills have been proposed and systematically compared (3, 7, 8). It emerged that interventions based on the repetition of isolated movements are less effective compared to interventions aimed at achieving a goal and acquiring skills that can become an integral part of a child’s daily routine (9). In these more effective interventions, the child is actively engaged in practicing age-appropriate tasks and movements that are relevant for him/her (7, 8, 10, 11). Of note, age plays an important role, since children under 8 years of age were more likely to succeed in improving their motor ability (3).

In the last decade, several task-specific interventions have been shown to be effective in children with unilateral CP. Among these, the bimanual therapy involved in the Hand-Arm Bimanual Intensive Therapy (Habit) has gained growing interest. It is an intervention where use of both hands in cooperation is required, aiming to increase functional independence during daily living. Starting from the HABIT, the HABIT Including Lower Extremities (HABIT-ILE) has been proposed as a form of bimanual training continuously incorporating also postural control and Lower Extremities function and tested in a randomized controlled trial in children with unilateral CP (12, 13). While both HABIT and HABIT-ILE were shown to be effective in improving Upper Extremities functions, interestingly, with respect to the HABIT, the HABIT-ILE induced larger functional improvements in activities of daily living involving the Lower Extremities (14).

Despite the possibility to improve upper extremities functions and daily living through these intensive trainings, there is a need to identify new forms of intervention that act more directly on brain plasticity to optimize neurorehabilitation effects especially in older children. This is particularly important when considering the cases in which the programs for improving fine and gross motor functions are delayed to a later, more chronic stage, with respect to the cerebral perinatal insult, due to the need of dealing with oral motor disorders or to other life-threatening conditions in the acute phase. Moreover, as the children grow, and brain plasticity slightly decreases, the needs and the desired goal of the individuals change, making it important to find a way to boost brain plasticity in combination with individualized rehabilitation training.

In this vein, vagus nerve stimulation (VNS) has been held as a tool to enhance the effect of motor rehabilitation in neurological disease like stroke (15). Animal models have demonstrated that pairing VNS with motor experience can result in cortical reorganization that can provide beneficial effects in stroke by driving specific forms of cortical plasticity (16). In rodent models of ischemic stroke, VNS combined with movement training significantly improved forelimb motor recovery and tripled the synaptic connectivity of motor cortex neurons compared with movement training alone. Interestingly, these benefits were stable after 6 weeks and the VNS-dependent recovery generalized also to similar untrained movements (17). In humans, the effects have been tested by Dawson et al. (18), who evaluated the clinical changes induced by VNS paired with physical rehabilitation in a pilot study with stroke patients. In this study, VNS was paired with several typical rehabilitative tasks over the course of 6 weeks of physical rehabilitation. At the conclusion of therapy, patients who received active VNS paired with rehabilitation demonstrated a significant increase of Upper Extremity Fugl–Meyer score compared with patients who received the same rehabilitation with sham VNS. The improved forelimb function persisted when evaluated 30 days after the conclusion of therapy, potentially suggesting a long-lasting recovery of function. In summary, researchers demonstrated a stronger enhancement in motor functions when motor rehabilitation was paired with VNS compared to when paired with sham VNS, in post-stroke patients. Unfortunately, VNS necessitates a costly, invasive surgical procedure.

In the last years, transcutaneous VNS (tVNS) has been proposed as a noninvasive and patient friendly method to stimulate the vagus nerve, through the skin, by delivering weak electric current to the sensory afferent fibers of the auricular, thick-myelinated, branch of the vagus nerve in the outer ear (19). Such stimulation activates the auricular branch of the vagus nerve and, via this pathway, the nuclei of the nerve located in the brainstem, enhancing brain GABA (20) and Noradrenaline (21) levels, which plays a pivotal role in brain plasticity (22). Since neuroimaging (23) and neurophysiological (20) studies suggested that the effect of tVNS on brain activity is quite similar to the effect induced by traditional, invasive VNS, tVNS has been proposed for the treatment of different clinical conditions including that consequent to stroke (24–30).

Considering the effectiveness of VNS in improving motor function following stroke in adults (18), the present study aims to investigate the effects of tVNS in the pediatric population with CP. The application of non-invasive brain stimulation (NIBS) techniques, such as tVNS, is encouraged also in pediatric populations (31, 32), with prior research indicating that NIBS modalities are generally safe and well tolerated also in children with brain injury (33–35).

With the present trial, we aimed at testing the boosting effects of tVNS when paired with an intensive bimanual training on motor functions in children and adolescents with CP.

Two groups of patients will receive active or sham tVNS paired with the HABIT-ILE protocol. In both groups, the intensive bimanual training will aim to the achievement of the goals that the children and their parents choose themselves, therefore tailored to patients’ needs and highly motivating.

We hypothesize, for both groups, an improvement after the training in the clinical assessments of hand motor ability and daily functioning. However, we also expect that patients treated with HABIT-ILE Training paired with active tVNS will show a greater and more lasting improvement compared to the patients receiving the HABIT-ILE protocol paired with sham tVNS (36). Furthermore, we anticipate that patients will not experience major adverse events due to tVNS (34).

## Design

This clinical trial represents one of the two Randomized Clinical Trials (RCT) of the project “Bottom-up and tOp-down neuromOdulation of motor plaSTicity in cerebral palsy” (BOOST; FRRB 3438840). The study is a randomized, double-blind, sham-controlled pre-post-test study involving 44 children and adolescents with CP. Patients will be treated in pairs, with a matching of motor deficit severity, IQ, or age, by meeting at least two out of these three criteria. Motor severity will be defined on the basis of the Manual Ability Classification System (MACS) level and classified as low to middle (I-II level) or as middle to high (II-III). With respect to age, two group levels will be identified for children (from 6 to 11 years old patients) and adolescents (12–17 years old patients). With respect to IQ, low IQ value (from 50 to 70) and high IQ value (> 71) will be distinguished. Each pair will be randomly assigned to the active or to the sham tVNS group. The randomization will be stratified based on IQ, MACS level, or age. As for the matching within each pair, at least two out of these three criteria must be encountered.

Both groups will undergo a bimanual training in an ecological and highly motivating environment while carrying out bimanual tasks, including recreational play activities during the application of the tVNS protocol. During the training, starting from a set of proposals the choice of the type of activities to be practiced in each session will be left to patients, boosting the motivational aspect of the training. This will also ensure an individualized and goal-directed approach. The therapists will monitor and modify the activities within each pair to ensure that the intervention maintains the quality of the individualized intervention. The positions performed during bimanual tasks and the activities of daily living will be designed to systematically engage postural control of the trunk and lower limbs according to Habit-ILE protocol. The training will be carried out by a therapist with expertise in maintaining the 1:2 ratios. Training activities will be carried out for 5 consecutive days for 3 h/day for 2 weeks (30 h of intervention overall). Children and adolescents will be assessed 1 or 2 days before the start of the treatment (T0), 1 or 2 days after the end of the treatment (T1), and at a follow-up after 3 months (T2).

The overall RCT will be structured as follows. In the first session, prior to the beginning of the training (T0), the RCT will include the administration of all clinical assessment measures and the patients will be asked to perform for 10 min an *ad-hoc* computer based Visuomotor task (please refer to the outcome sessions for more a detailed description). Then, patients will undergo the 10-day treatment. For each training day, the treatment will last 3 h. During the first 75 min of treatment, the active or sham tVNS (according to group allocation) will be delivered. Before starting, 20 min after and soon after the end of the stimulation, the vital parameters will be checked. After the end of the stimulation, patients will be also asked to rate the sensations experienced during the stimulation through Visual Analog Scales and through child-friendly Likert scales. Then, they will continue the training for the remaining session time without stimulation. Soon after the end of all the training sessions, patients will carry the T1 evaluation. As for the T0 session, this session will include the administration of the clinical assessment measures and the execution for 10 min of the Visuomotor task. Three months after the end of the training (T2), the follow up assessment will be conducted, following the same exact procedure of T0.

**Figure 1:**
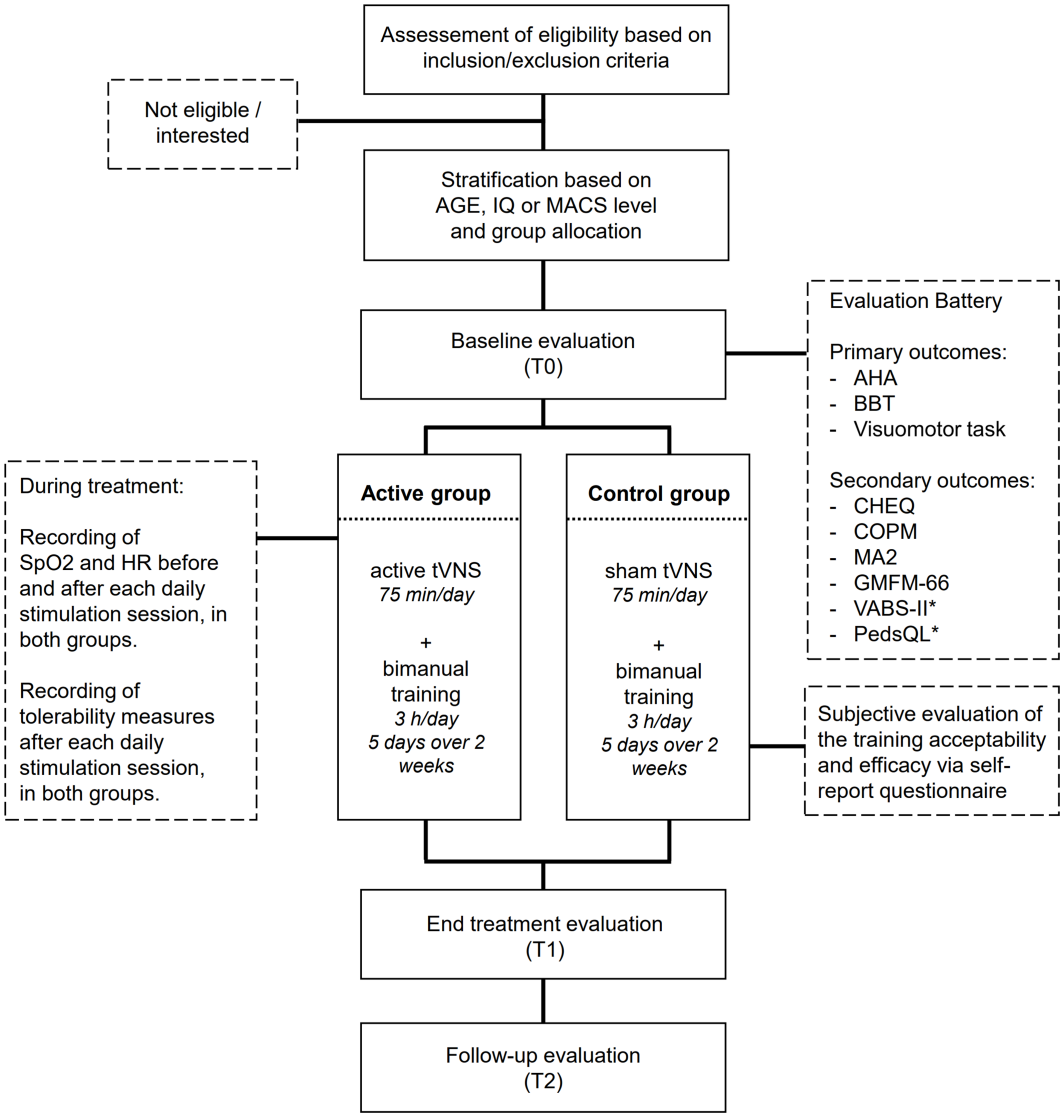
Flow chart of patient’s inclusion and experimental procedures. ^*^The PedsQL and VABS-II will be administered only at T0 and T2, while all other measures will be administered at all timepoints.

Primary outcomes will include the following clinical measures: Assisting Hand Assessment (AHA), Box and Block Test (BBT), and performance at the Visuomotor task. Secondary outcomes will include the scores at the following tests: Children’s Hand Experience Questionnaire (CHEQ), Canadian Occupational Performance Measure (COPM), Gross Motor Function Measure (GMFM-66); Melbourne Assessment-2 (MA2) scale, Vineland Adaptive Behavior Scale Version 2 (VABS II), and Pediatric Quality of Life Inventory (PEDS-QL). Vital parameters (i.e., oxygen saturation, SpO2, and Heart Rate, HR) and the scores at the questionnaires assessing stimulation-induced sensations will be also checked to assess the safety and the tolerability of the stimulation. Lastly, the feasibility and the acceptability of the training will be assessed by considering, respectively, the number of patients completing the training/the number of sessions for each patient and the response to *ad-hoc* questionnaires for the patients and their parents. All primary and secondary outcomes will be collected at each time point, except for the PEDS-QL and VABS II, which will be administered only at T0 and T2. Furthermore, vital cardiovascular parameters and the questionnaires assessing stimulation-induced sensations will be examined during each stimulation session. The acceptability questionnaires will be administered only after the end of the training (T1) (see Figure 1).

Each pair of patients will be randomly assigned to one of two groups (active vs. sham group) with an allocation ratio 1:1, through a computer-generated blocked randomization procedure conducted by independent personnel not directly involved in performing the intensive training. The patients and their parents, the personnel responsible for conducting the bimanual training and analyzing clinical data will be kept blind to the group allocation. Instead, the coordinator of the study and the personnel administering the tVNS will be “not blind.” With respect to the clinical assessment, the staff administering and scoring the scales will be blinded to group allocation. Questionnaire scores and neuropsychological tests will not contain personal information about the patients, who will be identified by an alphanumeric code. All records that contain names or other personal identifiers, such as informed consent forms, will be stored separately from study records identified by code number. In order to uphold the overall quality and credibility of the clinical trial, instances of code breaks should be limited to exceptional circumstances, where knowledge of the actual treatment is deemed absolutely necessary for the ongoing management of the patient. The intensive treatment will take place at the Scientific Institute, IRCCS E. Medea in Bosisio Parini (Lecco, Italy; study sponsor), Fondazione Mondino IRCCS in Pavia (Italy), and ASST Ospedali Civili in Brescia (Italy). The SPIRIT checklist used for protocol reporting can found in the Supplementary material.

## Patient selection

Male and female children and adolescents with a diagnosis of CP will be considered for eligibility if they are aged from 6 to 17 years. Inclusion criteria will comprise: the presence of clinical signs of unilateral or bilateral upper limb deficits, with a difference at least of 25% between the more affected and the less affected one at Box and Block test; a Magnetic Resonance Imaging confirmed diagnosis according to Surveillance of Cerebral Palsy (SCPE) criteria (37); MACS level I, II, III; Gross Motor Function Classification System level (GMFCS) I, II, III; Visual Function Classification System level (VFCS) between I, II, III; and IQ > 50. The exclusion criteria will include the presence of cochlear implant, cardiac pacemaker, ventriculoperitoneal shunt, neuro-stimulators, clips, fragments or metal splinters in the brain or skull except for titanium; treatments for spasticity or functional surgery of the upper limb in the previous 6 months or planned during the duration of the study, and uncontrolled epileptic seizures in the last 2 years. Before being included in the study, physicians will ensure thorough comprehension of the study’s purpose and design. The parents of the participants and each participant itself have to indicate their willingness to participate before being included.

## Outcome measures

**Figure 2:**
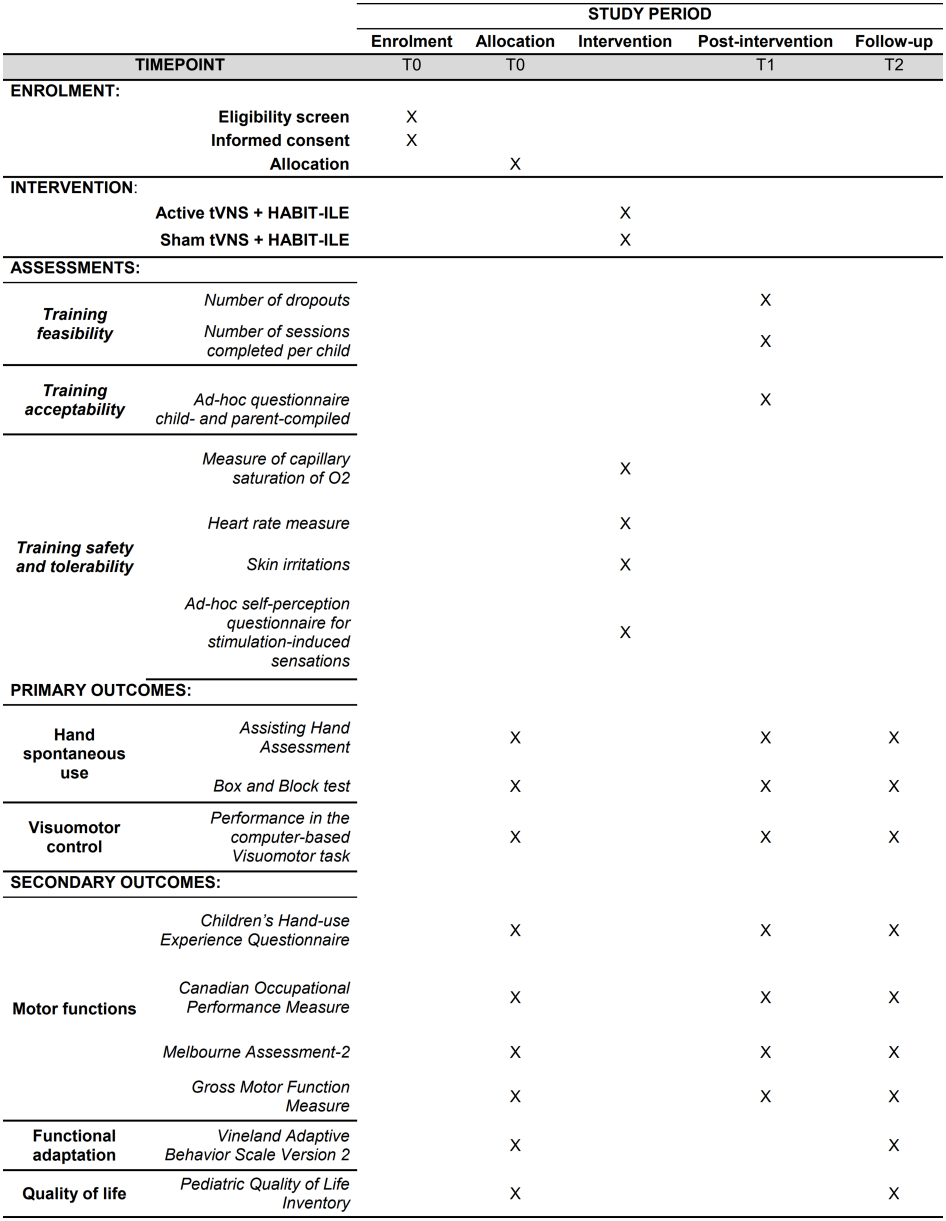
Schedule of enrolment, intervention, and assessment.

The primary outcomes consist of two validated measures of manual dexterity, namely the Assisting Hand Assessment (AHA), and the Box and Block test (BBT), and a pc-administered, *ad-hoc* visuomotor task for the evaluation of manual visuomotor control. Secondary outcomes encompass a comprehensive assessment of motor skills, functional adaptation, and quality of life. The following paragraphs provide a detailed description of both primary and secondary outcomes (Figure 2).

### Primary outcomes

#### Assisting hand assessment

This scale enables quantifying the assistance provided by the more affected hand to the less affected hand during bimanual activities. This observation-based, criterion-referenced assessment highlights a person’s typical performance, emphasizing practical functionality over maximal capacity, and serves as a reliable measure of change over time. The scale comprises 20 items, scored on a four-point Likert scale, from 1 to 4. The total score indicates how well the more affected hand is used as an assisting hand. A score of 20 means poor performance (the hand is not used as an assisting hand); a score of 80 means that the hand is used effectively. The results are converted for each of the three scales to logits by a Rasch analysis, on a 0–100 scale (with higher scores suggesting better use). This assessment has been shown to be responsive, reliable and valid for use in children with CP (38).

#### The box and block

This test is designed to measure manual dexterity. It is quick, simple, and cost-effective. It involves a box with a partition in the middle placed on a table, with a total of 150 blocks on one side of the partition. The score of the test is given by the number of blocks transported within a minute (39, 40) and it is performed with both the less and the more affected hands. The BBT provides a reliable and objective measurement of manual dexterity, making it valuable for evaluating functional outcomes and monitoring progress in rehabilitation programs over a short period of time (41).

#### The visuomotor task

This is an *ad hoc* computer-based task for the evaluation of manual visuomotor control. It involves a mouse click-and-drag operation where an object appears at the center of the screen. The objective is to drag and drop the object to the location indicated by a previously presented arrow, pointing toward a target object within a configuration of objects. Participants are required to focus on the direction of the arrow, swiftly and accurately moving the central object to its designated target location, by using a mouse with the less affected hand. This task enables the measurement of movement time (MT, in milliseconds; consisting in the time necessary to move the object in the target position); precision error (calculated as the distance, in pixels between the drop position of the object and the actual target position); the proportion of overtime errors (the percentage of trials in which responses are too slow). Smaller values of Movement time, Precision error and overtime errors indicate better performance.

### Secondary outcomes

#### The children’s hand-use experience questionnaire

The CHEQ is a parent report questionnaire that includes 27 bimanual activities (for example Cut up a pancake or other food easy to cut upon the plate). Each activity is rated on three scales measuring: (i) the perceived efficacy of the activity (“How do you think the child’s hand works?”) from 1 (meaning Bad/not used hand) to 4 (meaning Good efficacy); (ii) the amount of assistance and the time needed to perform the activity (“How much time does your child need to do the whole task, compared to peers?) from 1 (meaning Considerably longer) to 4 (meaning Equally long time compared to other peers); and (iii) child’s satisfaction with their performance (“Is your child bothered by his/her reduced hand/arm function during this activity?”) from 1 (corresponding to “It bothers him/her a lot”) to 4 (corresponding to “It does not bother him/her at all”). The questionnaire provides a result corresponding to the summary of the ratings. The results are converted for each of the three scales to logits by a Rasch analysis (42), on a 0–100 scale (with higher scores suggesting better performance/satisfaction).

#### The Canadian occupational performance measure

The COPM is a client-centered, semi-structured interview used in occupational therapy to identify the problems experienced by the patients. This interview engages the patient in recognizing daily occupations of importance that he/she wants to do, needs to do, or is expected to do but is unable to accomplish. Upon the identification of the problems experienced in a patient’s everyday-life activities, the child is asked to rate the importance of each activity in his/her life through a 10-point rating scale and then to select up to five problems to be addressed during the intervention. Lastly, the patient is asked to rate on a 10-point scale his/her own level of performance and satisfaction in performing that activity for each of the five problems (from 0, low performance or low satisfaction, to 10, high performance or high satisfaction) (43).

#### The Melbourne assessment-2

This scale allows evaluating the unimanual performance of both the more and less affected hand. It measures four elements of upper limb movement quality: movement range, accuracy, dexterity, and fluency. It consists of 14 test items that require children to interact (by reaching, grasping, releasing, and manipulating) with simple objects. Movement elements are scored on a three-, four-, or five-point scale according to specific criteria. Scores are arranged into the four sub-scales (movement range, accuracy, dexterity, and fluency) according to the element of movement being rated. A child’s total raw score for each sub-scale is converted to a percentage of the maximum possible score for that sub-scale, with higher scores indicating better performance. It is a valid and reliable measure for the assessment of the quality of upper limb movements in children with neurological conditions (44).

#### Gross motor function measure

It is a standardized observational tool used by healthcare professionals to evaluate and quantify the gross motor abilities and limitations of children with CP. It assesses 66 motor skills across five dimensions: lying and rolling, sitting, crawling and kneeling, standing, and walking, running, and jumping. Each skill is scored on a four-point scale, ranging from 0 (does not initiate) to 3 (performs fully). Particularly advantageous in time-limited clinical settings, the GMFM-66 remains a reliable and valid tool for evaluating gross motor skills, providing clinicians with essential information about patients’ capacities and tracks their progress effectively (45).

#### The Vineland adaptive behavior scale version 2

This is a tool designed to assess adaptive behavior in individuals from birth to age 90. It assesses 11 subdomains of adaptive behavior grouped into four domains: Communication, Daily Living Skills, Socialization, and Motor Skills. The sums of sub-dimension scores are standardized into domain standard scores. Sums of the domain standard scores are then standardized into the Adaptive Behavior Composite score ranging from 20 to 160 (mean = 100; standard deviation = 15). It has a proven track record of application in the evaluation of children with CP (46).

#### The pediatric quality of life inventory (cerebral palsy module)

The PedsQL is a widely employed, brief, and standardized self-reporting tool for assessing health-related quality of life in children and young individuals. The measure can be completed by parents (the Proxy Report) as well as by children and young people (the Self-Report), with versions available for children and young people with CP aged 5–7, 8–12, and 13–18 years old. Parent-rated versions are available for children aged 2–4, 5–7, 8–12, and 13–18 years old. The versions from 5 to 18 years comprise 35 items comprising seven dimensions: Daily Activities; School Activities; Movement and Balance; Pain and Hurt; Fatigue; Eating Activities; and Speech and Communication. For each item, consisting in everyday life action, it is required to indicate how much of a problem each item has been in the past month with response options from 0 (never a problem) to 4 (almost always a problem). The items are reverse scored and transformed to a 0–100 scale, with higher scores indicating better health-related quality of life. The PedsQL serves as a valuable resource for healthcare professionals in clinical settings, facilitating the assessment of various aspects of health-related quality of life in pediatric populations, including children with CP (47).

### Safety and tolerability measures of tVNS

Cardiovascular parameters such as capillary saturation of O2 (SpO2) and heart rate (HR) will be measured during a 2-min rest condition. For HR, the stimulation will be interrupted in case of values lower than 70 or higher than 120 beats per minutes (bpm) in 6–12 years old children and lower than 60 or higher than 100 bpm in 12–17 years old adolescents. Analogously, the stimulation will be interrupted for SpO2 values lower than 93%. These parameters will be checked in the first 2 min, and after 20 and 75 min of stimulation. Furthermore, potential skin irritations will be assessed through visual inspection during each session, before and after the application of tVNS, examining the skin at the electrode placement site for signs of redness, swelling, or rash.

The tolerability will be assessed through Visual Analog Scale by asking the level of discomfort experienced during the stimulation, and to rate on Child-friendly Likert scales the intensity of the following sensations: itching, pain, burning, heat, pinching, iron taste, and fatigue, among others. For each sensation the patients will be asked to express a value of perception strength that ranges from 0 (absence) to 4 (strong). Higher values will suggest stronger discomfort. Moreover, the questionnaire includes a final open-ended question to inquire about any other potential side effects they may have experienced during the stimulation. These measurements will be taken after the end of each stimulation session.

### Feasibility and acceptability of the training

Feasibility of the training will be assessed by considering (i) the number of patients who complete the 2-week training and (ii) the number of sessions completed per patient. These values, expressed as percentage and mean percentage will be extrapolated at T1. Higher values will indicate higher feasibility. Lastly, the acceptability will be assessed by asking the child/adolescent and his/her parents’ subjective evaluation of training accessibility and efficacy. The questionnaires will be adapted from the study of Butti et al. (48). Questions like “I find it difficult to motivate my child for doing the training”/“I find it difficult to start the training”; or “I would suggest this training to other people I know”/“I think other people I know would enjoy doing this training” will be rated from 1 (completely disagree) to 5 (completely agree). Response will be reversed in the negative items, so that higher scores will suggest more positive evaluation/higher acceptability.

## Interventional methods

### Transcutaneous nerve vagus stimulation

**Figure 3:**
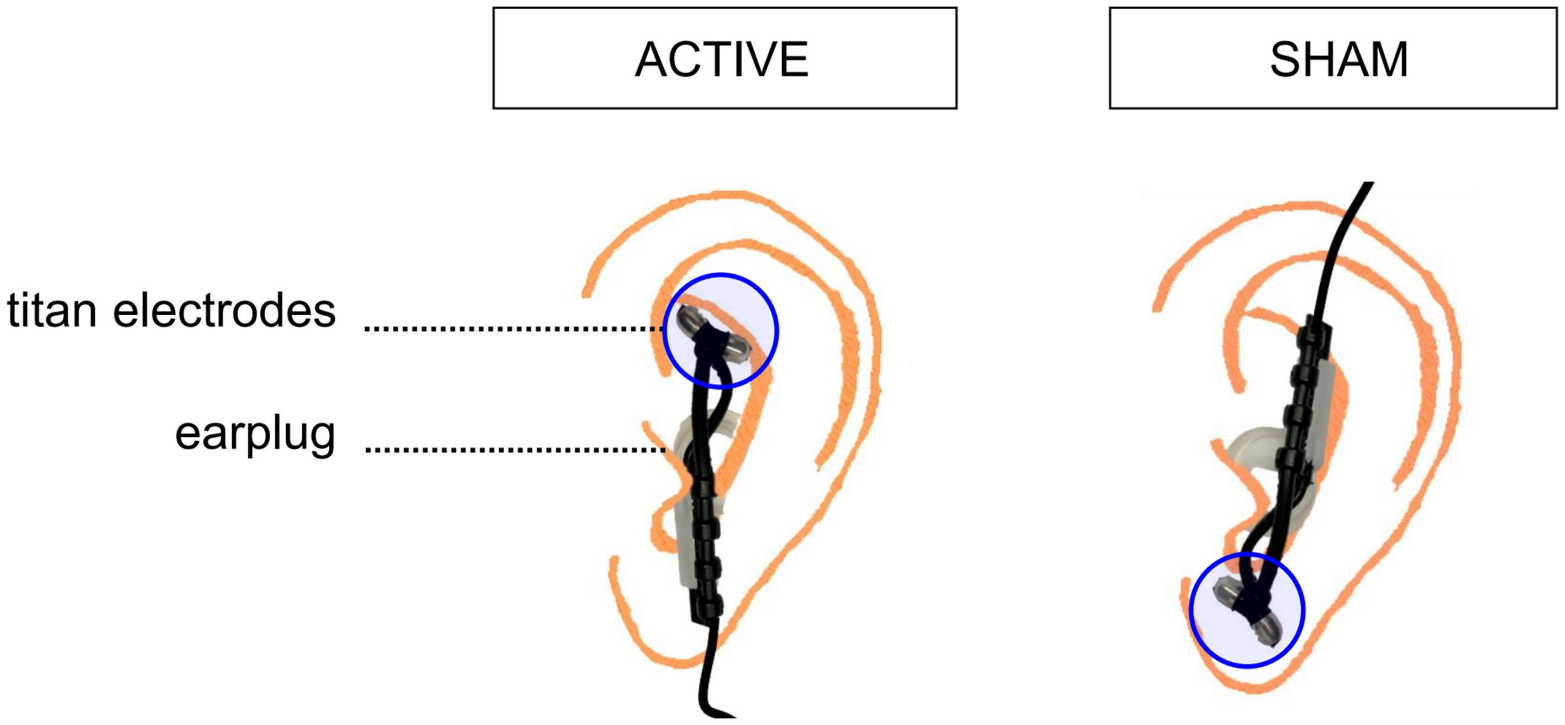
Schematic depiction of the electrode placements in the active and sham stimulation condition. To deliver active stimulation, the two titanium electrodes are placed on the outer auditory canal of the left ear. For sham stimulation, the two titanium electrodes are positioned on the center of the left earlobe.

Transcutaneous nerve vagus stimulation allows the non-invasive stimulation of the Vagus Nerve by delivering electrical pulses to the sensory afferent fibers of the auricular, thick-myelinated, branch of the vagus nerve in the outer ear. tVNS will be performed by using a CE-marked tVNS device tVNS®E (tVNS, technologies GMBH, Erlangen, Germany). It consists in a programmable stimulation unit connected to two titan ear electrodes that are mounted on a gel frame, allowing to generate and transfer electric impulses from the stimulation unit to the surface of the skin, where the electrodes are applied. The active tVNS will be applied through an active electrode placed on the cymba conchae (innervated by the auricular branch of the vagus nerve), while the sham stimulation will be administered by positioning the active electrode in correspondence of the earlobe, which is free of cutaneous vagus innervation (see Figure 3). Applying active stimulation in both the experimental and control groups, but at differently innervated positions of the ear skin, will allow for sham-control stimulation without hindering blindness to stimulation condition. To avoid cardiac effects, the electrodes will be applied to the left ear. The stimulation intensity will be set based on the perceptual threshold of the participants, corresponding to the perceived intensity and below the perception of pain, in order to make the stimulation tolerable. The device’s stimulation intensity ranges from 0.1 to 5 mA for individuals aged 3 years and older. However, in the present project the stimulation intensity will be constrained to range from a minimum of 0.5 mA to a maximum of 3 mA, falling within the range used in previous tVNS studies with pediatric populations (49). This will limit the risk of any major side effects (e.g., skin irritation) despite any sensorial deficits that may blur participants’ sensitivity to stimulation. In any case, the stimulation will be interrupted if participants would not tolerate the stimulation at the lowest predefined intensity. The intensity of the stimulation will be gradually increased to reach the intensity of stimulation with a ramping-up phase of 30 s. In both active and sham conditions, on (28 s) and off (32 s) periods of stimulation will be alternated, with pulses delivered every 200–300 μs at a frequency of 25 Hz. These are the most common parameters applied in tVNS studies, as outlined in a recent review on parameters settings for neurological and psychiatric disorders (50), and they are the default parameters of the CE-marked tVNS device used in this study. The tVNS, sham or active, will be applied for the first 75 min of the training, every day of the treatment. Given the duty cycle of alternating on and off periods, participants will receive 35 min of stimulation over a 75-min period in each session. Discontinuation to interventions will be contemplated in case of serious adverse effects, withdrawal of consent or significant changes in the participant’s health status, such as the development of any contraindication to the stimulation, that make continuing the intervention unsafe or inappropriate.

### HABIT-ILE protocol

Hand-Arm Bimanual Intensive Therapy Including Lower Extremities (12) is an intensive rehabilitation strategy of bimanual training that continuously incorporates postural control and lower extremity function (13). It is based on the assumption that lower extremity, postural function and interlimb coordination may benefit from being directly targeted in combination with the upper extremity. The Habit-ILE is based on motor learning and problem-solving, through the identification of problems, definition of tasks and progressive strategies to reach the objectives. It involves highly structured practice during which tasks requiring simultaneous control and coordination of upper and lower extremities are performed. In the Habit-ILE protocol, basic principles for inducing plastic change in the motor cortex are exploited, by means of repeated practice and gradual increase of task demand in a motivated and rewarding setting. In this vein, intervention will be based on child’s motor abilities (determined at baseline), age, interests and self-identified functional goals with parents’ advice (e.g., drinking by him/herself without spilling, tie the shoes, taking off/on a t-shirt, etc., as emerged also from the assessment with the COPM scale). According to the Habit-ILE protocol, tasks/activities will be made incrementally more challenging and the environment will be adapted to allow success of the child but still challenging the motor demands. Each child will start at a level, which he/she can easily achieve. At the lowest level, the more affected Upper Extremity will be used as a passive stabilizer, with progressive encouragement toward more complex (active) use. The tasks that will be performed include: (I) incremented table-top fine motor activities, performed in a seated position; (II) activities of daily living when sitting/standing/walking; and (III) gross motor play and physical activities. The involvement of the Lower Extremities in the different activities will also be progressed from sitting stable activities through an unsupported sitting and sitting on an unstable support, such as on a roller or a ball or a balance board, to dynamic activities, e.g., crawling, walking, running or jumping. More skilled/challenging Upper Extremity activities will be introduced in stable sitting, and then the Upper Extremity Activities that have been mastered will be carried out in more challenging conditions involving also Lower Extremities. During practice, ongoing feedback about performance will be provided. Activities will be presented as games and the environment will be arranged in such a way that children perceive it as fun (i.e., learning magical tricks, playing sports activities etc.), with the intent to increase motivation and compliance not only at the individual level, but also between the two participants. In this regard, the action of the therapists will focus on a few main aspects: fostering child-environment interaction (not only spatial environment, but also relational environment); promoting and supporting attention and motivation in the creation of the action plan so that both purely motor and sensory skills, as well as cognitive and emotional-relational skills, will be involved in the construction of the rehabilitation project. The involvement of experienced and *ad-hoc*-trained physical and occupational therapists is expected to ensure the consistent application of treatment methods, thereby enhancing adherence and maintaining fidelity to the intervention protocol. Importantly, the use of goal-directed activities chosen by the pair of children is expected to reduce frustration and provide the necessary motivation for repetitive bimanual activities. Participation in other clinical trials in the period spanning from 2 months before the beginning of this RCT until the follow up evaluation will be an exclusion criterion, in order to avoid interference with the study protocol and results. Standard care will be allowed.

## Sample size calculation

Using the GPower 3 software (51) with the “as in SPSS option” we estimated that, with a mixed design comprising the between group factor (active group vs. sham group) and the within variable (Pre T0-, post T1-, follow-up T2, numerator DF = 2), a sample of 22 subjects per group allows detecting, with a power of 0.80 and an alpha level set at 0.05, a between group difference between active and sham tVNS combined motor training of moderate to large effect size [f (U) = 0.35], which is expected to lead to a clinically-relevant improvement in motor activity and dexterity.

## Statistical analysis

Statistical analyses will be performed using STATISTICA 8.0 (StatSoftInc, Tulsa, Oklahoma). Demographic and clinical variables of the two groups of patients will be inspected through descriptive statistics. Independent sample *t*-test (two-tailed) and χ^2^ will be used to assess the differences between the experimental and control training groups at baseline for continuous and categorical variables, respectively, thus allowing us to verify successful randomization. The outcome measure data will undergo a normal distribution assessment using Kolmogorov–Smirnov tests. If the data adheres to a normal distribution, repeated measures ANOVAs will be employed to analyze differences between treatment group (active vs. sham) and across timepoints (T0-T1-T2) on the primary and secondary outcomes. Duncan *post hoc* tests will be performed to follow-up significant interactions and correct for multiple pair-wise comparisons. Conversely, for data not following a normal distribution, the Kruskal-Wallis H test or Friedman test will be applied. The exploratory analysis will center on investigating sex and age differences in treatment response, considering both primary and secondary outcomes. Furthermore, in light of findings on hemisphere-specific tVNS effects cortical excitability (20) and electrophysiological readiness potentials (52), we will conduct a sensitivity analysis. This analysis will examine potential differences in tVNS effects when administered ipsilateral or contralateral to the side of the most affected limb. Statistical significance will be denoted by *p* < 0.05. As to what concerns missing data, a modified intention-to-treat analysis approach will be adopted, including in the analyses all the participants who had completed the pre- and post-treatment evaluation sessions, even if they had not completed all the training sessions. No imputation of missing data, however, will be used considering the limited sample size and observation points.

## Data safety and management

The project involves the processing and storage of pseudonymized data, which will be archived in a centralized repository located on the servers of the study sponsor. Questionnaire scores and neuropsychological tests will not contain personal information about the participants, who will be identified by an alphanumeric code. All records that contain names or other personal identifiers, such as informed consent forms, will be stored separately from study records identified by code number. Access to the archives will be granted only through an authentication procedure and with appropriate usage rights. The project PI, if necessary, will carry out the unblinding procedure by accessing records containing names and personal identifiers.

## Discussion

Task-specific training, which are goal and functional oriented, are the most promising approach for the treatment of CP (9). Though, available evidence suggested that the effectiveness of these interventions decreases as the children grow (3). Thus, there is an urgent need to identify the most effective rehabilitation approaches that confer the greatest clinical gain, not only in the youngest, often “over-treated” children, but also in more chronic conditions, as for older children and adolescents with CP, using new forms of intervention that may adjuvate the rehabilitation by boosting brain plasticity.

The current trial aims at filling this gap, by proposing a protocol that can be individualized on patients’ needs and that will exploit neural plasticity as induced by tVNS. In this trial, 6–17 years old aged patients with unilateral or bilateral CP will be treated in pairs, with a matching of motor deficit severity, IQ, or age. Each pair will be randomly assigned to the active or to the sham tVNS group. During the application of the active or sham tVNS protocol, both groups will undergo a bimanual training according to the Habit-ILE protocol, which train bimanual tasks, including recreational play activities, while systematically engaging postural control of the trunk and lower limbs in an ecological and highly motivating environment. Training activities will be carried out for 2 consecutive weeks, 5 days per week, for 3 h/day, with an overall dose of 30 h of intervention. TVNS will be applied for the first 75 min in each session. As primary outcome measures, we will quantify the improvements in manual visuomotor control. Secondary outcomes will instead encompass a comprehensive assessment of motor skills, functional adaptation, and quality of life. Safety and tolerability will be evaluated too.

The study design provides for an active control group that will receive sham tVNS during participation to the training, thus accessing a treatment that represents the gold standard for CP. The comparison between the two groups will allow us to test the specific, modulatory effects on tVNS on the changes induced by the training. At the same time, it will allow us to test the transferability into everyday life adaptive behavior and quality of life.

Two major methodological issues should be considered and discussed when evaluating and interpreting the results of the protocol, in relation to the dosage of the intensive bimanual training and to the target population.

With respect to dosage of the intervention, there is not a consensus in the scientific community about the need for high-dose treatments and it is still unclear what can be considered a high-dose intervention. As argued by Hoare (53), therapeutic actions, including the selection and manipulation of tasks, practice, and feedback conditions, can improve or interfere with learning and generalization of skills. Dosage requirements appear to depend on the content and aims of therapy. Interventions that set functional goals and objectives and provide for actual practice of those goals lead to goal attainment at a lower dose than general upper limb motor training. While it is generally presumed that “more is better” (36, 54–56), some studies have found that “more” training did not lead to greater improvements (57–59). Accordingly, a systematic review of dose and intensity of practice for children with CP concluded that there was insufficient evidence to support high-dose interventions (60). In fact, a recent review of evidence in the unilateral population (61) has highlighted that children should practice goals for more than 30–40 h to improve general upper limb functions; however, to improve individual goals, children need to practice goals for more than 14–25 h. Given this evidence, we established the 30 h dosage of our treatment, in consideration of the cost and efforts of children and their families, as well as of the Health System.

With respect to the target population, children with clinical signs of unilateral or bilateral upper limb deficits will be involved. This could represent a limitation by considering that some of the assessment measures (such as the AHA) and the bimanual intensive training *per se* were developed for the evaluation of unilateral deficit. To overcome this limitation, we opted to include participants with bilateral impairment that also present a difference at least of 25% between the more affected and the less affected limb at BBT. This would ensure to include children with sufficiently different lateralized deficits (e.g., triplegic) and, thus, to easily identify the most compromised hand and the improvement of functionality in bimanual gestures. Of note, even if the inclusion of bilateral forms will increase the heterogeneity of the sample, it could support the generalization of the effects of the training to a wider sample of patients.

The use of NIBS in general, and of tVNS in particular, is gaining increasing consent as a tool to boost the effects of motor rehabilitation by exploiting brain plasticity, also with respect to pediatric age. Actually, the use of NIBS can be particularly advantageous in developmental age and in adolescents by benefitting from natural brain plasticity in younger children and by allowing to restore it in older ones.

However, an aspect deserving discussion pertains to the tolerability and safety of the stimulation. In general, tVNS is well tolerated and safe in both the pediatric and adult populations. To reduce any possible risks, participants will be thoroughly screened against the defined exclusion criteria to minimize the risks associated with the application of the stimulation. Moreover, to reduce potential effects on the heart, the electrode will be applied to the left ear, whose stimulation does not exert arrhythmic effects (19). The stimulation intensity will be set based on the perceptual threshold of the participants, corresponding to the perceived intensity and below the perception of pain. Setting the intensity below the pain threshold may lead to inconsistent stimulation across participants, resulting in variable outcomes that challenge data interpretation. This constitutes the main limitation of this approach. However, it also helps prevent dropout due to discomfort and promotes adherence to the training regime. Response variability according to inter-individual differences in tVNS responses will be also considered in order to identify best responses to tVNS with respect for example to gender, age and clinical impairments. Even if all recommended procedures will be followed, unwanted side-effects could be experienced by participants. As reported by the systematic review of a total of 51 studies published between the 2007 to the 2017 and involving a total of 1,322 participants (62), the most common effects could include tactile sensations and skin irritation under the electrode. Other less common sensations could include headache, vertigo, nausea, and nasopharyngitis. Very few studies reported cardiac side effects, that were transient and asymptomatic and gastrointestinal side effects. In each case, the occurrence of side effects and the intensity of each sensation will be accurately checked and safety parameters (SpO2 and HR) will be monitored.

Previous studies have documented the possibility to induce plasticity via VNS (63). Even if we will not directly test it in the current trial, we expect that the same effects should be induced through its non-invasive counterpart. Future studies should consider adopting neurophysiological measures by assessing, for example, the way in which such a training could affect activity and plasticity of the motor cortex, for example measuring cortico-spinal excitability, cortical inhibition and long-term potentiation induced by paired associative stimulation, via transcranial magnetic stimulation, or brain rhythms, via electroencephalographic recording.

## Dissemination

Bimonthly meetings will be scheduled to monitor the progress of enrolment and data analysis. The findings of this study will be released to the participating physicians, referring physicians, patients and the general scientific community. Relevant results will be also disseminated through patients’ association websites, the official project’s website, and through dedicated brochures for families, describing the methodologies and results of the treatments to be distributed to rehabilitation and care centers. Results will be presented at academic and/or clinical conferences. The study will result in at least one peer-reviewed publication of quantitative findings in a national or international medical journal. CONSORT guidelines (64) will be followed when reporting this RCT. The authors will adhere to principles of transparency and reproducibility in research by following the procedures presented in this study protocol and by sharing research material and anonymized dataset of this study in public repositories (https://osf.io/).

## Conclusion

The proposal for this study stems from evidence regarding the efficacy of task-oriented bimanual training (30 h) in children with CP, the growing experiences made in the field of neuromodulation through brain stimulation in the adult population affected by adult stroke, and the possibility to obtain, through NIBS, similar effects to the ones obtained with invasive VNS. The results of this project will provide evidence on the possibility to translate this technique to developmental age and to enhance the effects of motor rehabilitation of patients with CP using tVNS. We expect patients in the real-tVNS group to show greater and longer-lasting improvements on the assessed outcome measures following the motor training as compared to patients in the sham group, indicating stimulation-induced cumulative effects in boosting the training improvement. The proposed rehabilitation treatment model, indeed, includes intensive, but short and repeatable, treatment cycles, innovative NIBS methodologies, possibility of working in pairs, even in groups, and in an ecological environment. This would provide a short-term, effective rehabilitation approach that may improve the quality of life of children, decreasing medicalization and promoting their socialization and participation.

## Ethics statement

The studies involving humans were approved by Comitato Etico I.R.C.C.S. Eugenio Medea. Prot. N. 66/22-CE; Comitato Etico Pavia, Prot. N. 0014336/23; Comitato Etico Brescia, Prot. N. 5673. The studies will be conducted in accordance with the local legislation and institutional requirements. Written informed consent for participation in this study will be provided by the participants’ legal guardians/next of kin. Before the study begins, all participants and their parents or guardians will receive both oral and written descriptions of the protocols, including the potential risks and benefits. Three different versions of these descriptions (in Italian) will be provided to ensure comprehension: one for the parents, one for adolescents (12–17 years old), and one for children (6–11 years old). Written informed consent will be obtained from all parents. Minors aged 12 or older will be asked to give their written assent for participation. One copy of the consent form will be given to the participant, and another will be kept by the experimenter. Each recruitment center will be responsible for collecting and storing the informed consent of every patient recruited within their facility. Important protocol modifications will be reported to the Fondazione Regionale per la 1 Ricerca Biomedica (FRRB) and clinicaltrials.gov, and approved by the Ethics Committees.

## Author contributions

VO: Writing – original draft, Conceptualization. VG: Writing – original draft, Conceptualization. AM: Writing – review & editing, Conceptualization. AC: Writing – review & editing. RB: Writing – review & editing, Funding acquisition. SO: Writing – review & editing. EF: Writing – review & editing, Funding acquisition. AM: Writing – review & editing. JG: Writing – review & editing. LP: Writing – review & editing. CM: Writing – review & editing. MA: Writing – review & editing. ZC: Writing – review & editing, Funding acquisition. CU: Writing – original draft, Conceptualization. AF: Writing – original draft, Conceptualization, Funding acquisition.
